# Supplementary material for: Intraindividual Variability Matters for the Correspondence Between Subjective Arousal, Valence, and Physiological Responses
Source: Psychophysiology. 2026 Jan 15;63(1):e70224. doi: 10.1111/psyp.70224 (PMC12808866; doi:10.1111/psyp.70224)
Supplement: Supplementary file 1 — Data S1: psyp70224‐sup‐0001‐Supinfo.docx. [file PSYP-63-e70224-s001.docx]

**Supplementary material.**

1. Linear mixed models of the effects of Category on SCR and Startle.
2. Comparison between time-series- and amplitude-based RSMs of SCR.
3. Consistency of the time-series- and amplitude-based RSM of SCR across tasks and samples.
4. Representational Similarity Analysis for the association between SCR and arousal-based models during the PPV task in the replication sample
5. Representational Similarity Analysis for the association between SCR and arousal-based models during the PSL task in the replication sample.
6. Representational Similarity Analysis for the association between SCR and arousal-based models during the Imagery task in the replication sample.
7. Representational Similarity Analysis for the association between startle and valence-based models during the PPV task in the replication sample.
8. Representational Similarity Analysis for the association between startle and valence-based models during the PSL task in the replication sample.
9. Representational Similarity Analysis for the association between startle and valence-based models during the Imagery task in the replication sample.
10. Visual depiction of RSMs construction and analyses performed.
11. Linear mixed models of the effects of Category on SCR and Startle

To ensure that the effects of the current data sets are in line with previous studies, showing affect-specific modulations in SCR and startle responses^1–3^, we carried out Linear Mixed Effects Models, using the lme4-package^4^. The categorical independent variable was valence category (i.e., pleasant, unpleasant or neutral; neutral was used as reference), whereas the dependent variable was z-transformed SCR and EMG amplitudes (discovery sample) or log-transformed mean phasic driver (replication sample) as well as t-transformed startle magnitudes (in both samples).

*Discovery Sample*

A significant main effect of picture valence emerged in both startle and SCR. For startle, the effect was significant (*F*(2, 20,077) = 268.48, *p* < .001), with a pattern of negative valence > neutral valence > positive valence (all pairwise comparisons *p* < .001). Similarly, for SCR, a significant effect was observed (*F*(2, 37,505) = 65.78, *p* < .001), with the valence gradient of negative > positive > neutral (all pairwise comparisons *p* < .002). startle responses aligned with the expected valence gradient, while SCR responses followed the expected arousal gradient.

*Replication Sample*

Skin Conductance Response. In the passive picture viewing (PPV) task, both pleasant, *t*(2,063)=7.25, *p* < .001, and unpleasant images, *t*(2,063)=6.42, *p* < .001, evoked larger SCRs compared to neutral images. In the passive sound listening (PSL) task, pleasant sounds evoked larger SCR than neutral ones, *t*(2,133)=4.32, *p* < .001, but no such effect was found for unpleasant relative to neutral sounds, *t*(2,133)=1.57, *p* = .12. In line with the PPV task, in the imagery task, both pleasant, *t*(2,133)=3.52, *p* < .001, and unpleasant scripts evoked larger SCR than neutral ones, *t*(2,133)=5.09, *p* < .001.

Startle eye blink response. In the PPV task, although pleasant and neutral pictures evoked comparable responses, *t(*1,088) =-0.75, *p* = .45, unpleasant images produced larger startle blink responses than neutral ones, *t(*1,088)=2.18, *p* = .029. In the PSL task, pleasant sounds evoked smaller responses than neutral ones, *t(*1,088)=-2.15, *p* = .032, but no significant difference was found for unpleasant relative to neutral sounds, *t*(1,088)=1.56, *p* = .117. In the Imagery task, unpleasant scripts evoked larger startle blink responses than neutral ones, *t*(1,107)=2.93, *p* = .003, but no differences emerged between pleasant and neutral scripts, *t*(1,107)=1.54, *p* = .12.

2. Comparison between time-series- and amplitude-based/phasic driver RSMs of SCR.

In our previous study^5^, the RSMs of SCR were derived from the time-series of the SCR from 1 to 4 seconds after stimulus onset. This information was, however, unavailable in the discovery sample. Thus, in a preliminary step we tested the similarity between time-series- and amplitude-based and phasic driver RSMs of SCR in the replication sample. If both RSMs showed high correspondence, it would indicate that similar processes underlie both parameters, and their usage may be interchangeable. If that would not be the case, it would indicate that they are influenced by different factors. In the latter case, to determine which parameters to use, the consistency across tasks and samples was further tested (Suppl. S3).

For the PPV task, the similarity index (rho= -.04) did not surpass the permutation-based significant threshold (rho= .07, p = .786, BF_10_ = 0.001). A similar pattern was found in the PSL (similarity index: rho= -.02; threshold: rho= .07, p = .658, BF_10_ = 0.005) and Imagery tasks (similarity index: rho= -.18; threshold: rho= .14, p = .96, BF_10_ = 0.002). These results indicate that time-series and amplitude parameters of the SCR are influenced by different factors.

Figure S1 depicts the RSM of time-series- (below the diagonal) and amplitude-based (above the diagonal) RSMs as well as the permutation test, comparing their correspondence in each of the three tasks.


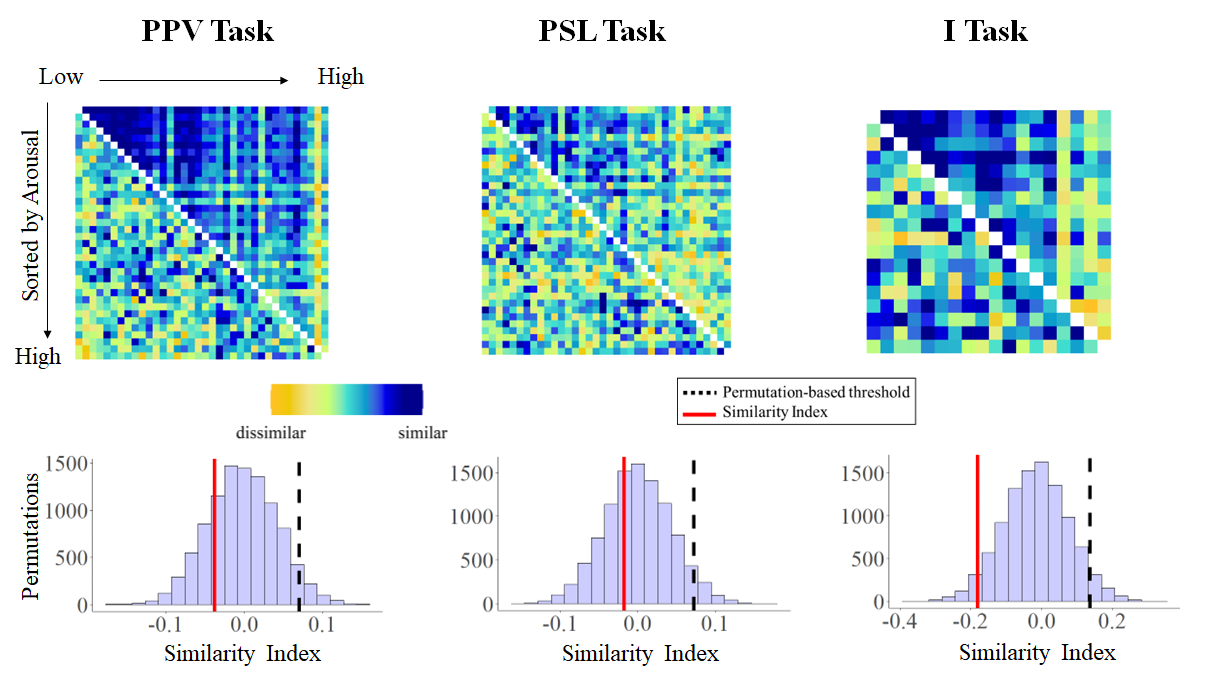


Figure S1. Results of the permutation tests comparing the RSMs derived from time-series and amplitudes of the SCR. In the upper part, the RSMs for both time-series (below the diagonal) and amplitudes (above the diagonal) are depicted for each task, PPV (right), PSL (middle), and Imagery tasks (left). In the lower part, the results of the permutation tests are depicted whereby the distribution of the permutation tests and the permutation-based threshold (black dotted line) as well as the similarity between both RSMs (red solid line) are represented. Results revealed no significant correspondence between RSMs across tasks, indicating that they are influenced by different factors.

3. Consistency of the time-series- and amplitude-based RSM of SCR across tasks and samples.

Because no correspondence was found between time-series- and amplitude-based RSMs of SCR, in the following step, we examined the consistency of both parameters by comparing RSMs from the PPV and PSL tasks. For the time-series RSMs, results revealed no similarity between RSMs (rho= .01, threshold = .11, p = .84; BF_10_ = .002). For the amplitude RSMs, however, a significant correspondence was observed, rho = .18, threshold: rho = .11, p <.001, BF_10_ = 6.69). The amplitude-based RMSs not only showed consistency between tasks within the same sample, but also significant correspondence when comparing the RSMs of the PPV and PSL task with the PPV task of the discovery sample (PPV discovery sample and PPV replication sample: rho = .39, threshold: rho = .11, p <.001, BF_10_ > 100; PPV discovery sample and PSL replication sample: rho = .26, threshold: rho = .10, p <.001, BF_10_ > 100).

Figure S2 depicts the RSM of the amplitude-based across tasks and samples as well as the permutation test, comparing their correspondence.


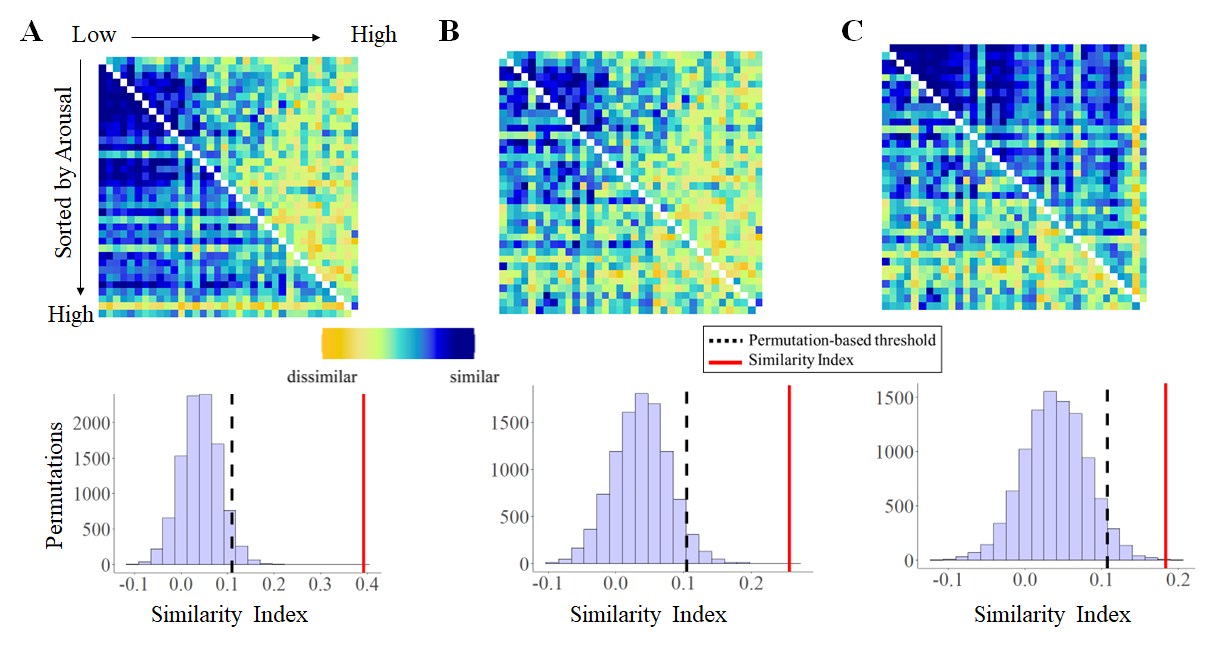


Figure S2. Results of the permutation tests comparing the RSMs derived from amplitudes of the SCR across tasks and samples. A) In the upper part, the RSMs for the SCR from the PPV of the discovery sample (above the diagonal) and from the PPV of the replication sample (below the diagonal). B) In the upper part, the RSMs for the SCR from the PPV of the discovery sample (above the diagonal) and from the PSL of the replication sample (below the diagonal). C) In the upper part, the RSMs for the SCR from the PPV (above the diagonal) and from the PSL of the replication sample (below the diagonal). In all sections (A, B, and C), the lower part depicts the results of the permutation tests whereby the distribution of the permutation tests and the permutation-based threshold (black dotted line) as well as the similarity between both RSMs (red solid line) are represented. Results revealed evidence for a correspondence between RSMs across tasks, and samples, indicating that they reflect similar processes.

4. Representational Similarity Analysis for the association between SCR and arousal-based models during the PPV task in the replication sample

In the PPV task of the replication sample (N = 64), a similar pattern of results was observed as in the discovery sample. Both averaged RSMs of SCR (i.e., considering and dismissing intraindividual variability) were related to each other, rho = .46, p <.001, BF10 >100, but showed different association with the NN and inverted AK models of arousal. For the averaged RSM of SCR dismissing intraindividual variability, decisive evidence for a positive association with the NN model of arousal was found, rho = .43, p <.001, BF_10_ >100, and strong evidence for a relationship with the inverted AK model of arousal, rho = .13, p = .015, BF_10_ = 71.27). Importantly, when models were regressed out, and the correspondence between the models residuals and the averaged RSM of SCR was tested, it was found decisive evidence for a correspondence between the averaged RSM of SCR dismissing intraindividual variability and the NN model (rho = .39, p <.001, BF_10_ >100), and moderate evidence for no correspondence with the inverted AK model (rho = -.01, p =.79, BF_10_ =0.14; Figure S3A-C). These results again indicate that when intraindividual variability is dismissed, the overall pattern of the SCR is better explained by the NN model.

When the averaged RSM of SCR considering intraindividual variability was compared to the models of arousal, a positive association with both the NN and inverted AK model was observed (NN model: rho = .10, p =.012 , BF_10_ = 3.18; inverted AK model: rho = .59, p <.001, BF_10_ >100). However, regression analysis revealed decisive evidence for an association with the inverted AK model (rho = .55, p <.001, BF_10_ >100), and for a lack of a positive association with the NN model (rho = -.06, p = .998 , BF_10_ = 0.001; Figure 2-E).

We further tested which of the averaged RSMs of SCR was a better representative of the individual RSMs of SCR. Results revealed decisive evidence for a positive relationship between the individual RSMs of SCR and the averaged RSM of SCR dismissing intraindividual variability, Mean = 0.09, t(58)= 6.07, p <.001, BF_10_ >100, and with the averaged RSM of SCR considering intraindividual variability, Mean = 0.17, t(58)= 9.67, p <.001, BF_10_ >100. Most importantly, when models were regressed out, the individual RSM were uniquely related to averaged RSM of SCR considering intraindividual variability (averaged RSM of SCR dismissing intraindividual variability: Mean = -0.001, t(58)= -0.1, p = .92, BF_10_ = 0.14; averaged RSM of SCR considering intraindividual variability: Mean = 0.14, t(58)= 9.77, p <.001, BF_10_ >100; Figure S3G), indicating that participants more often show a pattern of SCR similar to the averaged RSM of SCR considering intraindividual variability.

Individual level analysis revealed anecdotal evidence for a positive relationship between both the NN and the inverted AK and the individual RSM of SCR (NN model: Mean = .02, t(58)=2.06, p = .04, BF_10_ = 1.01; inverted AK model: Mean = .12, t(58)= 7.01, p <.001, BF_10_ >100) but regression analysis showed decisive evidence for the correspondence with the inverted AK model (Mean = .11, t(58)= 6.24, p <.001, BF_10_ >100) and no correspondence with the NN model (Mean = .008, t(58)= 0.69, p = .48, BF_10_ = 0.17; Figure S3H). The evidence for the association between the individual RSM of SCR and the inverted AK model remained after controlling for time, valence-based, and arousal-based categorical, as well as valence-based dimensional models (time-based model: Mean = .12, t(58)= 6.88, p <.001, BF_10_>100; valence-based categorical model: Mean = .08, t(58)= 7.00, p <.001, BF_10_ >100; arousal-based categorical model: Mean = .12, t(58)= 6.81, p <.001, BF_10_ >100; valence-based dimensional model: Mean = .11, t(58)= 6.9, p <.001, BF_10_ >100; Figure S3I).


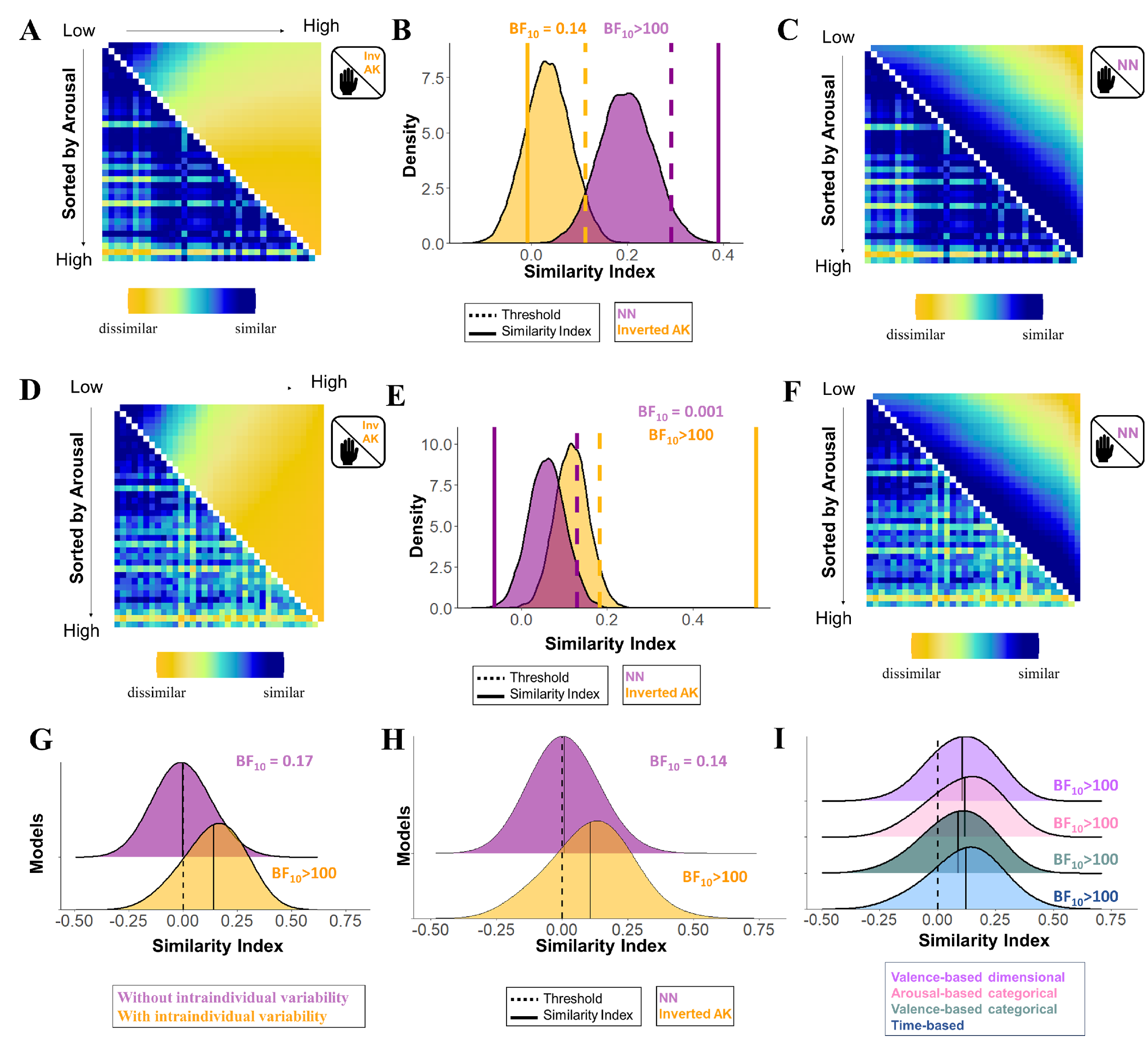


Figure S3. Association between the representational similarity matrices (RSMs) of subjective arousal based on the inverted Anna Karenina (inv AK) and Nearest Neighbors (NN) models (after controlling for the shared similarities), and the RSMs of the skin conductance response (SCR) dismissing (A-C) and considering (D-F) intraindividual variability (replication sample, N= 64, PPV task). A) Averaged RSM of SCR dismissing intraindividual variability (lower diagonal) and averaged RSM of subjective arousal based on the inverted AK model (upper diagonal). B) Results of the permutation test. C) Averaged RSM of SCR dismissing intraindividual variability (lower diagonal) and averaged RSM of subjective arousal based on the NN model (upper diagonal). D) Averaged RSM of SCR considering intraindividual variability (lower diagonal) and averaged RSM of subjective arousal based on the inverted AK model (upper diagonal). E) Results of the permutation test. F) Averaged RSM of SCR considering intraindividual variability (lower diagonal) and averaged RSM of subjective arousal based on NN model (upper diagonal). G) Distribution of the association between individual RSMs of SCR with the unique contribution of the averaged RSMs of SCR disregarding (purple) and considering (yellow) intraindividual variability. H) Distribution of the association between individual RSMs of SCR and unique contribution of the individual RSM of arousal based on the inverted AK and NN models. I) Distribution of the association between individual RSMs of SCR and individual RSMs of subjective arousal based on the inverted AK model after controlling for other models (i.e., time-based, valence-based categorical, arousal-based categorical, valence-based dimensional models).

5. Representational Similarity Analysis for the association between SCR and arousal-based models during the PSL task

In the PSL task of the replication sample (N = 64), both averaged RSMs of SCR (i.e., considering and dismissing intraindividual variability) were not related to each other, rho = .04, p =.13 , BF_10_ = 0.43. For the averaged RSM of SCR dismissing intraindividual variability, decisive evidence for a positive association with the NN model of arousal was found, rho = .33, p <.001, BF_10_ >100, and decisive evidence for a lack of a positive relationship with the inverted AK model of arousal was observed, rho = -.14, p = .99, BF_10_ < 0.001). Importantly, when models were regressed out, and the correspondence between the model residuals and the averaged RSM of SCR was tested, strong evidence was found for a correspondence between the averaged RSM of SCR dismissing intraindividual variability and the NN model (rho = .36, p = .012, BF_10_ = 12.37), and decisive evidence for a lack thereof with the inverted AK model (rho = -.24, p =.99, BF_10_ < 0.001; Figure S4 A-C). These results again indicate that when intraindividual variability is dismissed, the overall pattern of the SCR is better explained by the NN model.

When the averaged RSM of SCR considering intraindividual variability was compared to the models of arousal, moderate evidence for a lack of association between the averaged RSM of SCR and the NN model was found (rho = .06, p =.076, BF_10_ = 0.295) whereas decisive evidence for a correspondence with the inverted AK model was observed (rho = .45, p <.001 , BF_10_ > 100). Regression analysis corroborated the exclusive correspondence with the inverted AK model (inverted AK model: rho = .45, p <.001, BF_10_ >100; NN model: rho = -.08, p = .993 , BF_10_ = 0.004; Figure S4 D-F).

We further tested which of the averaged RSMs of SCR (with or without considering intraindividual variability) was a better representative of the individual RSMs of arousal. Results revealed strong evidence for a lack of a positive relationship between the individual RSMs of SCR and the averaged RSM of SCR dismissing intraindividual variability, Mean = 0.003, t(60)= 0.35, p =.72, BF_10_ = 0.14, but decisive evidence was found for an association with the averaged RSM of SCR considering intraindividual variability, Mean = 0.12, t(60)= 8.9, p <.001, BF_10_ > 100. The results did not change when models were regressed out (averaged RSM of SCR dismissing intraindividual variability: Mean = -0.007, t(60)= -.88, p = .38, BF_10_ = 0.20; averaged RSM of SCR considering intraindividual variability: Mean = 0.12, t(60)= 9.03, p <.001, BF_10_ > 100; Figure S4G), indicating that participants more often show a pattern of SCR similar to the averaged RSM of SCR considering intraindividual variability.

Individual level analysis showed decisive evidence for a positive relationship between the inverted AK model and the individual RSM of SCR (Mean = .07, t(60)=4.01, p <.001, BF_10_ > 100), and evidence for the lack of a relationship with the NN model (Mean = .01, t(60)=1.25, p = .21, BF_10_ = 0.29). Further regression analysis validated these results (inverted AK model: Mean = .07, t(60)= 4.48, p <.001, BF_10_ > 100; NN model: Mean = -.007, t(60)= -0.65, p = .51, BF_10_ = 0.17, Figure S4H). The evidence for the association between the individual RSM of SCR and the inverted AK model was further observed after controlling for time, valence-based, and arousal-based categorical, as well as valence-based dimensional models (time-based model: Mean = .06, *t*(60)= 3.75, p <.001, BF_10_ > 100; valence-based categorical model: Mean = .07, *t*(60)= 4.22, p <.001, BF_10_ > 100; arousal-based categorical model: Mean = .06, *t*(60)= 3.82, p <.001, BF_10_ =76; valence-based dimensional model: Mean = .07, *t*(60)= 4.57, p <.001, BF_10_ >100; Figure S4I).


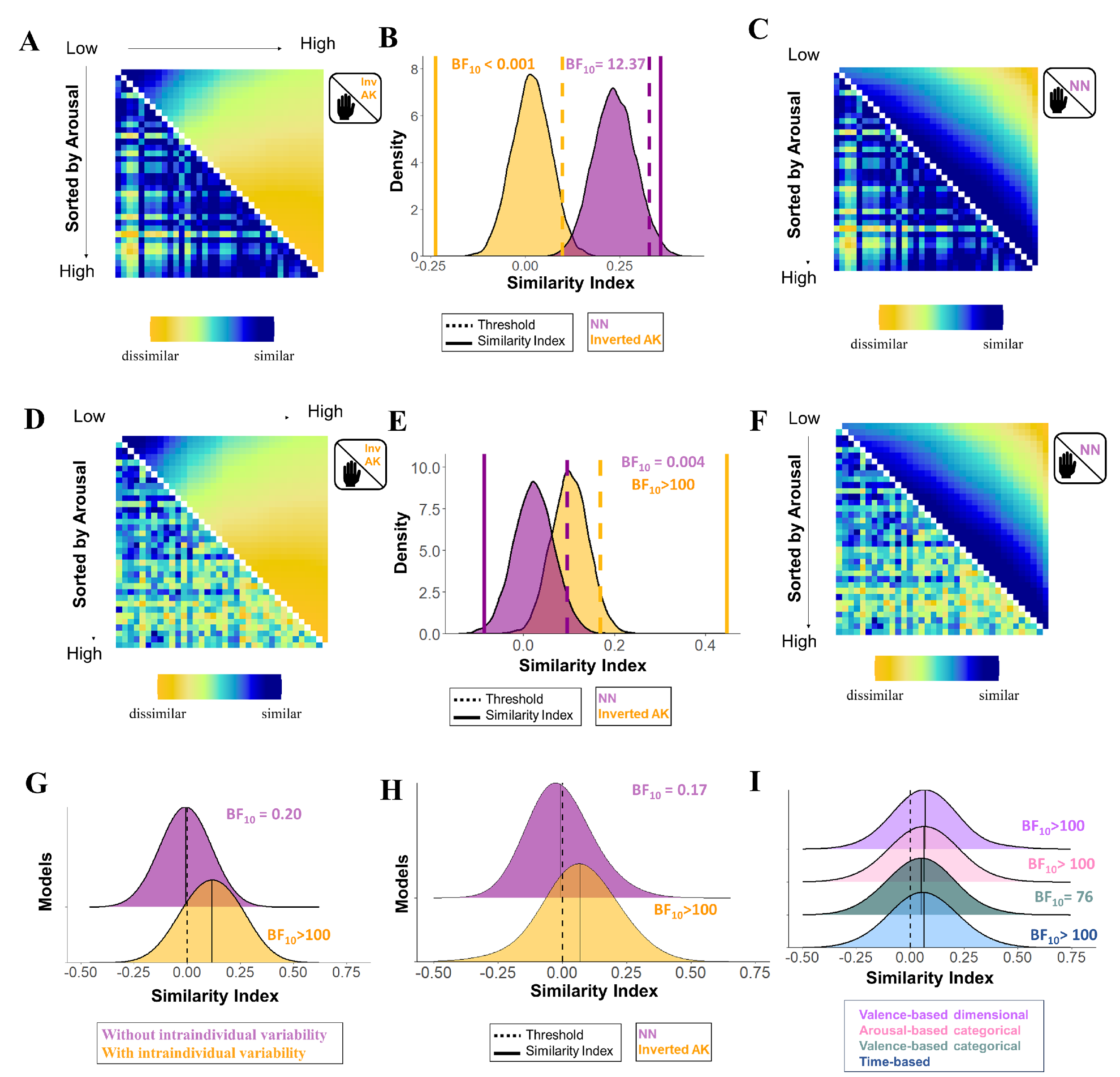
Figure S4. Association between the representational similarity matrices (RSMs) of subjective arousal based on the inverted Anna Karenina (inv AK) and Nearest Neighbors (NN) models (after controlling for the shared similarities), and the RSMs of the skin conductance response (SCR) dismissing (A-C) and considering (D-F) intraindividual variability (replication sample, N= 64, PSL task). A) Averaged RSM of SCR dismissing intraindividual variability (lower diagonal) and averaged RSM of subjective arousal based on the inverted AK model (upper diagonal). B) Results of the permutation test. C) Averaged RSM of SCR dismissing intraindividual variability (lower diagonal) and averaged RSM of subjective arousal based on the NN model (upper diagonal). D) Averaged RSM of SCR considering intraindividual variability (lower diagonal) and averaged RSM of subjective arousal based on the inverted AK model (upper diagonal). E) Results of the permutation test. F) Averaged RSM of SCR considering intraindividual variability (lower diagonal) and averaged RSM of subjective arousal based on NN model (upper diagonal). G) Distribution of the association between individual RSMs of SCR with the unique contribution of the averaged RSMs of SCR disregarding (purple) and considering (yellow) intraindividual variability. H) Distribution of the association between individual RSMs of SCR and unique contribution of the individual RSM of arousal based on the inverted AK and NN models. I) Distribution of the association between individual RSMs of SCR and individual RSMs of subjective arousal based on the inverted AK model after controlling for other models (i.e., time-based, valence-based categorical, arousal-based categorical, valence-based dimensional models).

6. Representational Similarity Analysis for the association between SCR and arousal-based models during the Imagery task.

In the Imagery task of the replication sample (N = 64), both averaged RSMs of SCR (i.e., considering and dismissing intraindividual variability) were related to each other, rho = .47, p <.001 , BF_10_ > 100. For the averaged RSM of SCR dismissing intraindividual variability decisive evidence for a positive association with the NN model of arousal was found, rho = .32, p <.001, BF_10_ >100, and moderate evidence for a positive relationship with the inverted AK model of arousal, rho = 0.21, p = .02, BF_10_ = 3.86). When models were regressed out, and the correspondence between the model residuals and the averaged RSM of SCR was tested, it was found moderate evidence for a lack of correspondence between the averaged RSM of SCR dismissing intraindividual variability and the NN model (rho = .28, p = .30, BF_10_ = 0.22), and anecdotal evidence for an association with the inverted AK model (rho = .16, p =.092, BF_10_ = 1.16; Figure S5 A-C).

When the averaged RSM of SCR considering intraindividual variability was compared to the models of arousal, strong evidence for a lack of correspondence between the averaged RSM of SCR and the NN model of arousal was observed (rho = .01, p =.387 , BF_10_ = 0.052), whereas decisive evidence for a correspondence with the inverted AK model of arousal was found (rho = .65, p <.001 , BF_10_ > 100). Regression analysis corroborated these findings (NN model: rho = -.09, p = .998, BF_10_ = 0.042; inverted AK model: rho = .61, p <.001, BF_10_ >100; Figure S5 D-F).

We further tested which of the averaged RSM of SCR was a better representative of the individual RSMs of SCR. Results revealed anecdotal evidence for a positive relationship between the individual RSMs of SCR and the averaged RSM of SCR dismissing intraindividual variability, Mean = 0.04, t(60)= 2.17, p =.03, BF_10_ = 1.25, but decisive evidence for an association with the averaged RSM of SCR considering intraindividual variability, Mean = 0.12, t(60)= 4.93, p <.001, BF_10_ >100. The results were even more pronounced when models were regressed out (averaged RSM of SCR dismissing intraindividual variability: Mean = -0.02, t(60)= -1.55, p = .11, BF_10_ = 0.46; averaged RSM of SCR considering intraindividual variability: Mean = 0.12, t(60)= 5.77, p <.001, BF_10_ >100; Figure S5 G), indicating that participants more often show a pattern of SCR similar to the averaged RSM of SCR that considers intraindividual variability.

In line with the average analysis, in the individual level analysis decisive evidence in favor of a positive association between SCR and the inverted AK model (Mean = .10, t(60)=4.15, p <.001, BF_10_ > 100) and a lack thereof with the NN model was found (Mean = -.01, t(60)=-1.34, p = .18, BF_10_ = 0.33). Further regression analysis validated these results (NN model: Mean = -.02, t(60)= -1.91, p = .06, BF_10_ = 0.76, inverted AK model: Mean = .11, t(60)= 4.59, p <.001, BF_10_ > 100; see Figure S5 D). The evidence for the association between the individual RSM of SCR and the inverted AK model was still observed after controlling for, valence-based, and arousal-based categorical, as well as valence-based dimensional models (valence-based categorical model: Mean = .10, t(60)= 4.25, p <.001, BF_10_ > 100; arousal-based categorical model: Mean = .1, t(60)= 4.13, , p <.001, BF_10_ > 100; valence-based dimensional model: Mean = .07, t(60)= 4.11, p <.001, BF_10_ >100).


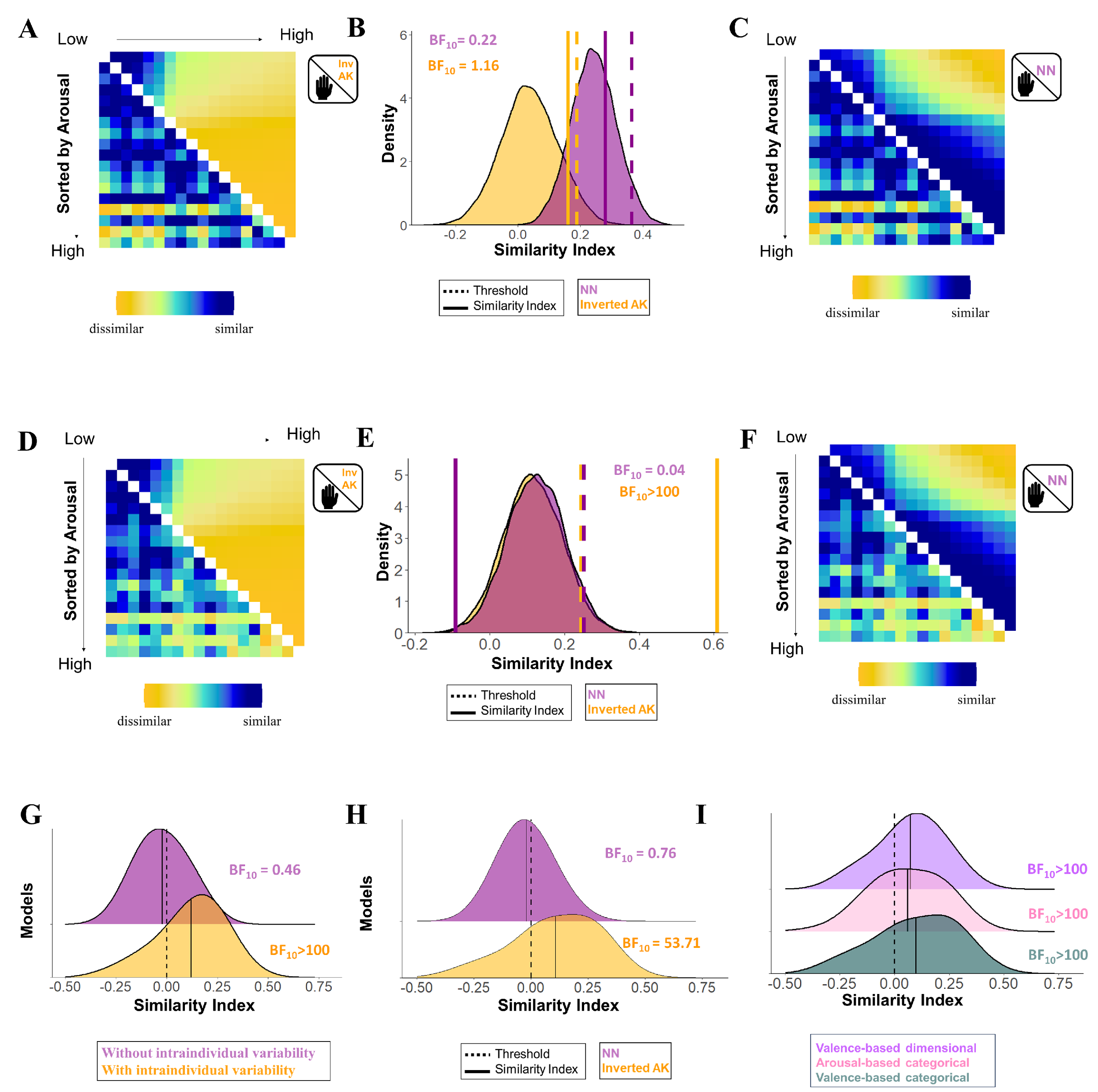
Figure S4. Association between the representational similarity matrices (RSMs) of subjective arousal based on the inverted Anna Karenina (inv AK) and Nearest Neighbours (NN) models (after controlling for the shared similarities), and the RSMs of the skin conductance response (SCR) dismissing (A-C) and considering (D-F) intraindividual variability (replication sample, Imagery task, N= 64). A) Averaged RSM of SCR dismissing intraindividual variability (lower diagonal) and averaged RSM of subjective arousal based on the inverted AK model (upper diagonal). B) Results of the permutation test. C) Averaged RSM of SCR dismissing intraindividual variability (lower diagonal) and averaged RSM of subjective arousal based on the NN model (upper diagonal). D) Averaged RSM of SCR considering intraindividual variability (lower diagonal) and averaged RSM of subjective arousal based on the inverted AK model (upper diagonal). E) Results of the permutation test. F) Averaged RSM of SCR considering intraindividual variability (lower diagonal) and averaged RSM of subjective arousal based on NN model (upper diagonal). G) Distribution of the association between individual RSMs of SCR with the unique contribution of the averaged RSMs of SCR disregarding (purple) and considering (yellow) intraindividual variability. H) Distribution of the association between individual RSMs of SCR and unique contribution of the individual RSM of arousal based on the inverted AK and NN models. I) Distribution of the association between individual RSMs of SCR and individual RSMs of subjective arousal based on the inverted AK model after controlling for other models (i.e., valence-based categorical, arousal-based categorical, valence-based dimensional models).

7. Representational Similarity Analysis for the association between startle and valence-based models during the PPV task.

For the PPV task, both averaged RSMs of startle (i.e., considering and dismissing intraindividual variability) were related to each other, rho = .26, p <.001 , BF_10_ = 15.64. For the averaged RSM of startle dismissing intraindividual variability, moderate evidence for a positive association with the NN model of valence was found, rho = .22, p = .009, BF_10_ = 4.61, and strong evidence for a lack of a positive relationship with the AK model of valence, rho = .09, p = .31, BF_10_ = 0.06. When models were regressed out, and the correspondence between the model residuals and the averaged RSM of startle was tested, it was found moderate evidence for correspondence between the averaged RSM of startle dismissing intraindividual variability and the NN model of valence (rho = .22, p = .007, BF_10_ = 5.56), and strong evidence for a lack of correspondence with the AK model of valence (rho =.09, p =.31, BF_10_ = 0.06; Figure S6 A-C).

When the averaged RSM of startle considering intraindividual variability was compared to the models of valence, evidence for a lack of correspondence between the RSM of startle and the NN and AK models of valence was found at an average level (NN model: rho = .13, p = .08 , BF_10_ = 0.83; AK model: rho = .10, p =.28, BF_10_ = 0.05; regression analysis: NN model: rho = .12, p = .08 , BF_10_ = 1.01; AK model: rho = .10, p =.27, BF_10_ = 0.05; Figure S4 D-F).

When the averaged RSMs of startle were compared to the individual RSMs, results revealed anecdotal evidence for a positive relationship between the individual RSMs of startle and the averaged RSM of startle dismissing intraindividual variability, Mean = 0.03, t(60)= 2.47 p =.01, BF_10_ = 2.31, but decisive evidence for an association with the averaged RSM of startle considering intraindividual variability, Mean = 0.11, t(60)= 8.38, p <.001, BF_10_ >100. The results were even more noticeable when models were regressed out (averaged RSM of startle dismissing intraindividual variability: Mean = 0.00, t(60)= 0.02, p = .98, BF_10_ = 0.14; averaged RSM of startle considering intraindividual variability: Mean = 0.11, t(60)= 7.58, p <.001, BF_10_ >100; Figure S6 G), indicating that participants more often show a pattern of startle similar to the averaged RSM of startle considering intraindividual variability.

At an individual level, no evidence for a positive relationship between individual RSM of startle and models of valence was found (NN model: Mean = .01, t(60)=0.69, p = .49, BF_10_ = 0.16; AK model: Mean = .01, t(60)= 0.53, p =.59, BF_10_ =0.17; regression analysis: NN model: Mean = .02, t(60)=1.45, p = .15, BF_10_ = 0.38; AK model: Mean = .02, t(60)= 0.99, p =.32, BF10=0.22; Figure S6 I). Moreover, similar evidence for a lack of association between the individual RSM of startle and the AK model was found after controlling for time-based (Mean = .01, t(60)= 0.31, p = .75, BF_10_ =0.14), valence-based categorical (Mean = .01, t(60)= 0.48, p =.63, BF_10_ = 0.16), arousal-based categorical models (Mean = .01, t(60)= 0.49, p =.62, BF_10_ = 0.16) and arousal-based dimensional model (Mean = .01, t(60)= 0.43, p = .67, BF_10_ = 0.15).


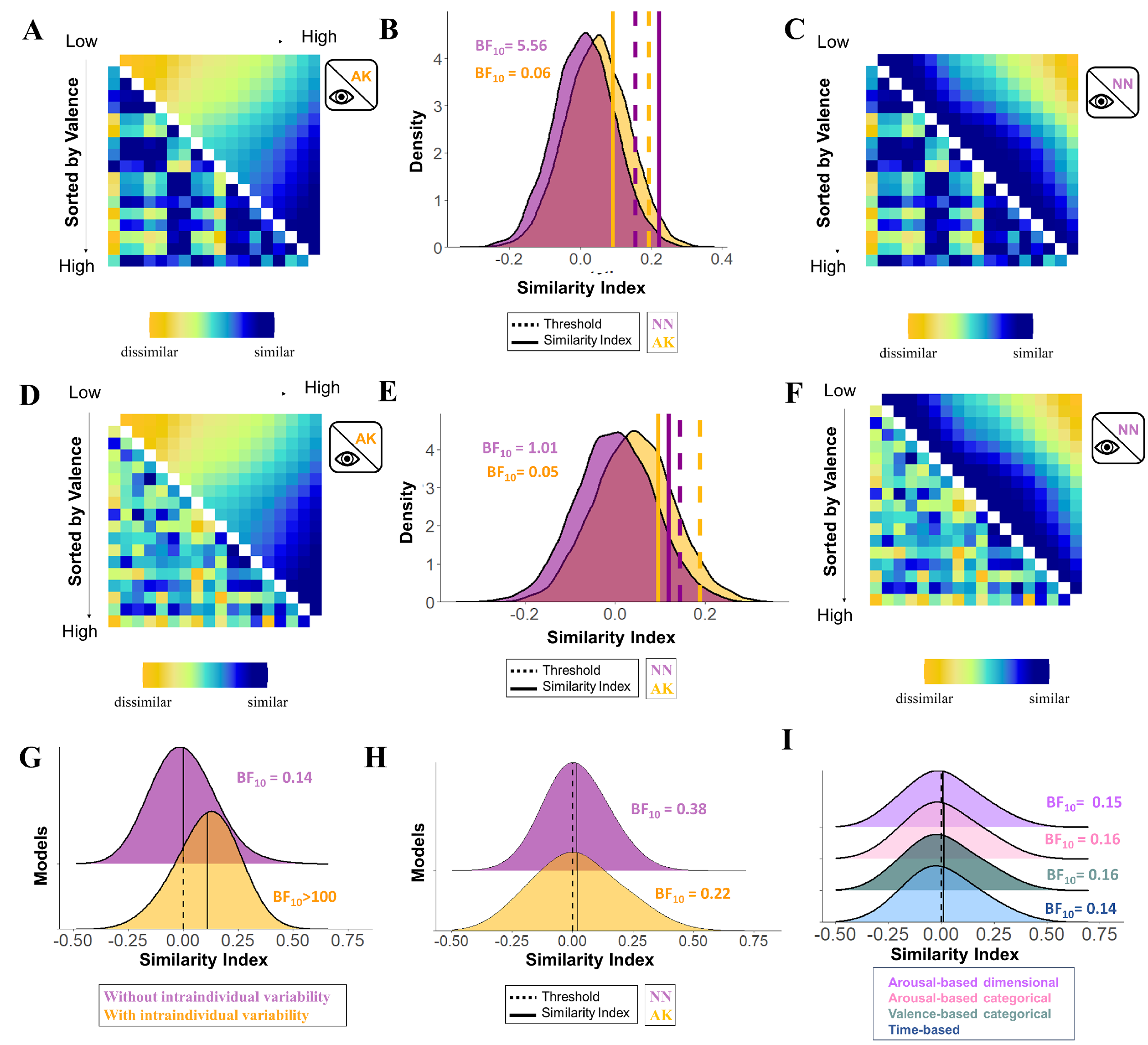


Figure S6. Association between the representational similarity matrices (RSMs) of subjective valence based on the Anna Karenina (AK) and Nearest Neighbours (NN) models (after controlling for the shared similarities), and the RSMs the startle eye blink response dismissing (A-C) and considering (D-F) intraindividual variability (replication sample, PPV task, N= 64). A) Averaged RSM of startle dismissing intraindividual variability (lower diagonal) and averaged RSM of subjective valence based on the AK model (upper diagonal). B) Results of the permutation test. C) Averaged RSM of startle dismissing intraindividual variability (lower diagonal) and averaged RSM of subjective valence based on the NN model (upper diagonal). D) Averaged RSM of startle considering intraindividual variability (lower diagonal) and averaged RSM of subjective valence based on the AK model (upper diagonal). E) Results of the permutation test. F) Averaged RSM of startle considering intraindividual variability (lower diagonal) and averaged RSM of subjective valence based on NN model (upper diagonal). G) Distribution of the association between individual RSMs of startle with the unique contribution of the averaged RSMs of startle disregarding (purple) and considering (yellow) intraindividual variability. H) Distribution of the association between individual RSMs of startle and unique contribution of the individual RSM of valence based on the AK and NN models. I) Distribution of the association between individual RSMs of startle and individual RSMs of subjective valence based on the AK model after controlling for other models (i.e., time-based, valence-based categorical, arousal-based categorical, valence-based dimensional models).

8. Representational Similarity Analysis for the association between startle and valence-based models during the PSL task

For the PSL task of the replication sample (N = 64), both averaged RSMs of startle (i.e., considering and dismissing intraindividual variability) were related to each other, rho = .3, p <.001, BF_10_ = 12.39. For the averaged RSM of startle dismissing intraindividual variability, anecdotal evidence for a lack of a positive association with the NN model of valence was found, rho = .14, p = .063, BF_10_= 0.80, and strong evidence for lack of a positive relationship with the AK model of valence, rho = -.001, p = .65, BF_10_= 0.02. When models were regressed out, and the correspondence between the model residuals and the averaged RSM of startle was tested, it was found anecdotal evidence for a lack of correspondence between the averaged RSM of startle dismissing intraindividual variability and the NN model of valence (rho = .14, p = .16, BF_10_= 0.93), and strong evidence for a lack of correspondence the AK model of valence (rho =-.002, p =.67, BF_10_= 0.02; Figure S7 A-C).

When the averaged RSM of startle considering intraindividual variability was compared to the models of valence, anecdotal evidence for a positive association between the AK, but not the NN model, and the averaged RSM of startle was found (AK model: rho = .23, p =.018, BF_10_= 1.53; NN model: rho = -.09, p = .874 , BF_10_= 0.01). These results were further verified in the regression analysis (NN model: rho = -.10, p =.887, BF10=0.002; AK model: rho = .23, p =.018, BF_10_= 1.68; Figure S7 A-C).

We also tested which of the averaged RSM of startle was a better representative of the individual RSMs of startle. Results revealed anecdotal evidence for a positive relationship between the individual RSMs of startle and the averaged RSM of startle dismissing intraindividual variability, Mean = 0.03, t(60)= 2.21 p =.03, BF10 = 1.35, but decisive evidence for an association with the averaged RSM of startle that considering intraindividual variability, Mean = 0.12, t(60)= 7.91, p <.001, BF_10_>100. The results were even more pronounced when models were regressed out (averaged RSM of startle dismissing intraindividual variability: Mean = -0.003, t(60)= -0.19, p = .84, BF_10_= 0.84; averaged RSM of startle considering intraindividual variability: Mean = 0.12, t(60)= 7.52, p <.001, BF_10_>100; Figure S7 G), indicating that participants more often show a pattern of startle similar to the averaged RSM of startle considering intraindividual variability.

At an individual level, results revealed evidence for a lack of a positive association of the individual RSM of the startle with both the NN model (Mean = -0.01, t(60)=-1.03, p = .31, BF_10_= 0.23) nor the AK model of valence (Mean = .034, t(60)=1.91, p =.059, BF_10_= 0.77; regression analysis: NN model: Mean = -.01, t(60)=-0.82, p = .41, BF_10_= 0.19; AK model: Mean = .034, t(60)= 2.09, p =.04, BF_10_=1.06; Figure S7 D). Moreover, similar evidence between the individual RSM of startle and the AK model was found after controlling for time-based (Mean = .032, t(60)= 1.87, p = .066, BF_10_=0.71), valence-based categorical (Mean = .034, t(60)= 1.96, p =.058, BF_10_= 0.83), and arousal-based categorical models (Mean = .034, t(60)= 1.93, p =.058, BF_10_= 0.79) and arousal-based dimensional model (Mean = .035, t(60)= 2.03, p = .04, BF_10_= 0.95; Figure S7I).


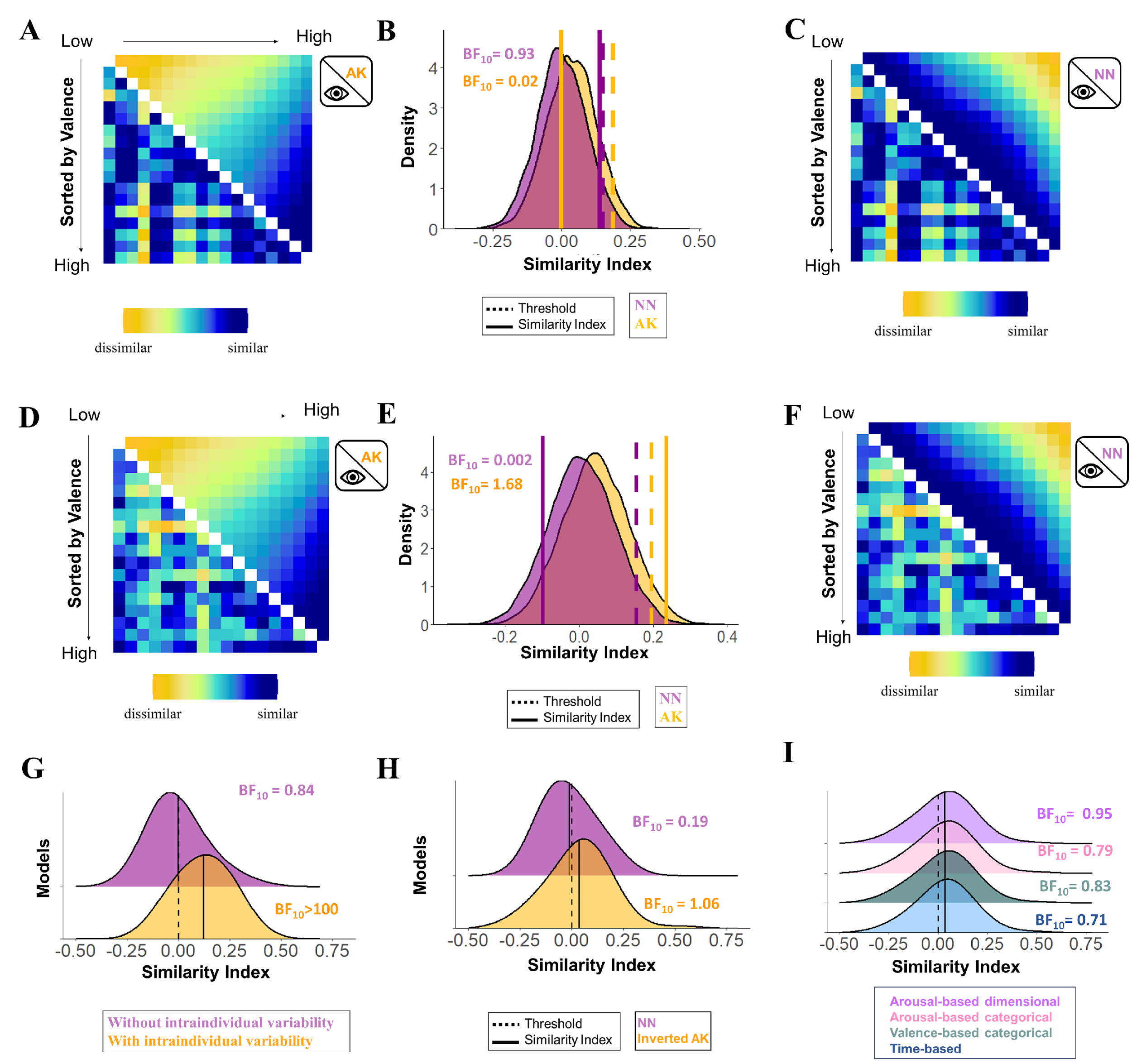


Figure S7. Association between the representational similarity matrices (RSMs) of subjective valence based on the Anna Karenina (AK) and Nearest Neighbours (NN) models (after controlling for the shared similarities), and the RSMs the startle eye blink response dismissing (A-C) and considering (D-F) intraindividual variability (replication sample, PSL task, N= 64). A) Averaged RSM of startle dismissing intraindividual variability (lower diagonal) and averaged RSM of subjective valence based on the AK model (upper diagonal). B) Results of the permutation test. C) Averaged RSM of startle dismissing intraindividual variability (lower diagonal) and averaged RSM of subjective valence based on the NN model (upper diagonal). D) Averaged RSM of startle considering intraindividual variability (lower diagonal) and averaged RSM of subjective valence based on the AK model (upper diagonal). E) Results of the permutation test. F) Averaged RSM of startle considering intraindividual variability (lower diagonal) and averaged RSM of subjective valence based on NN model (upper diagonal). G) Distribution of the association between individual RSMs of startle with the unique contribution of the averaged RSMs of startle disregarding (purple) and considering (yellow) intraindividual variability. H) Distribution of the association between individual RSMs of startle and unique contribution of the individual RSM of valence based on the AK and NN models. I) Distribution of the association between individual RSMs of startle and individual RSMs of subjective valence based on the AK model after controlling for other models (i.e., time-based, valence-based categorical, arousal-based categorical, valence-based dimensional models).

9. Representational Similarity Analysis for the association between startle and valence-based models during the Imagery task.

For the Imagery task, both averaged RSMs of startle (i.e., with and without intraindividual variability) were related to each other, rho = .30, p <.001 , BF_10_= 19.91, For the averaged RSM of startle dismissing intraindividual variability, moderate evidence for a lack of a positive association with the NN model of valence was found, rho = .09, p = .17, BF_10_= 0.20, and decisive evidence for lack of a positive relationship with the AK model of valence, rho = -.06, p = .81, BF_10_ <.001). When models were regressed out, and the correspondence between the model residuals and the averaged RSM of startle was tested, it was found moderate evidence for a lack of correspondence between the averaged RSM of startle dismissing intraindividual variability and the NN model of valence (rho = .1, p = .13, BF_10_= 0.30), and decisive evidence for a lack of correspondence the AK model of valence (rho =-.08, p =.84, BF_10_= 0.001 Figure S8 A-C).

When the averaged RSM of startle considering intraindividual variability was compared to the models of valence, no evidence for a correspondence between the averaged RSM of startle and the NN and AK models was found e (NN model: rho = .11, p = .17 , BF_10_= 0.51; AK model: rho = .19, p =.06, BF_10_= 0.41; regression analysis: NN model: rho = .08, p = .222 , BF_10_= 0.42; AK model: rho = .17, p =.078, BF_10_= 0.36; Figure S8 D-F).

When the averaged RSMs of startle were compared to the individual RSMs, results revealed anecdotal evidence for a lack of positive relationship between the individual RSMs of startle and the averaged RSM of startle dismissing intraindividual variability, Mean = 0.03, t(61)= 1.87, p =.06, BF_10_= 0.71, but decisive evidence for an association with the averaged RSM of startle considering intraindividual variability, Mean = 0.12, t(61)= 6.69, p <.001, BF_10_>100. The results remained when models were regressed out (averaged RSM of startle dismissing intraindividual variability: Mean = -0.007, t(61)= -0.54, p = .60, BF_10_= 0.16; averaged RSM of SCR considering intraindividual variability: Mean = 0.12, t(61)= 6.81, p <.001, BF_10_>100; Figure S8 G), indicating that participants more often show a pattern of SCR similar to the averaged RSM of SCR considering intraindividual variability.

At the individual level evidence for a lack of a positive association between individual RSMs of startle and models of valence was found (NN model: Mean = .01, t(61)=1.12, p = .26, BF_10_= 0.21; AK model: Mean = .02, t(60)= 0.96, p =.39, BF_10_= 0.25; regression analysis: NN model: Mean = .01, t(61)= 1.23, p = .22, BF_10_= 0.28; AK model: Mean = .02, t(61)= 0.83, p =.40, BF_10_=0.19; Figure S7 H). Moreover, no evidence for the association between the individual RSM of startle and the AK model was found after controlling for time-based (Mean = .02, t(61)= 1.00, p = .32, BF10=0.23), valence-based categorical (Mean = .02, t(61)= 1.00, p =.32, BF_10_= 0.21), arousal-based categorical models (Mean = .02, t(61)= 1.01, p =.32, BF_10_= 0.23), and arousal-based dimensional model (Mean = .02, t(61)= 0.96, p = .34, BF_10_= 0.21 Figure S8 I).


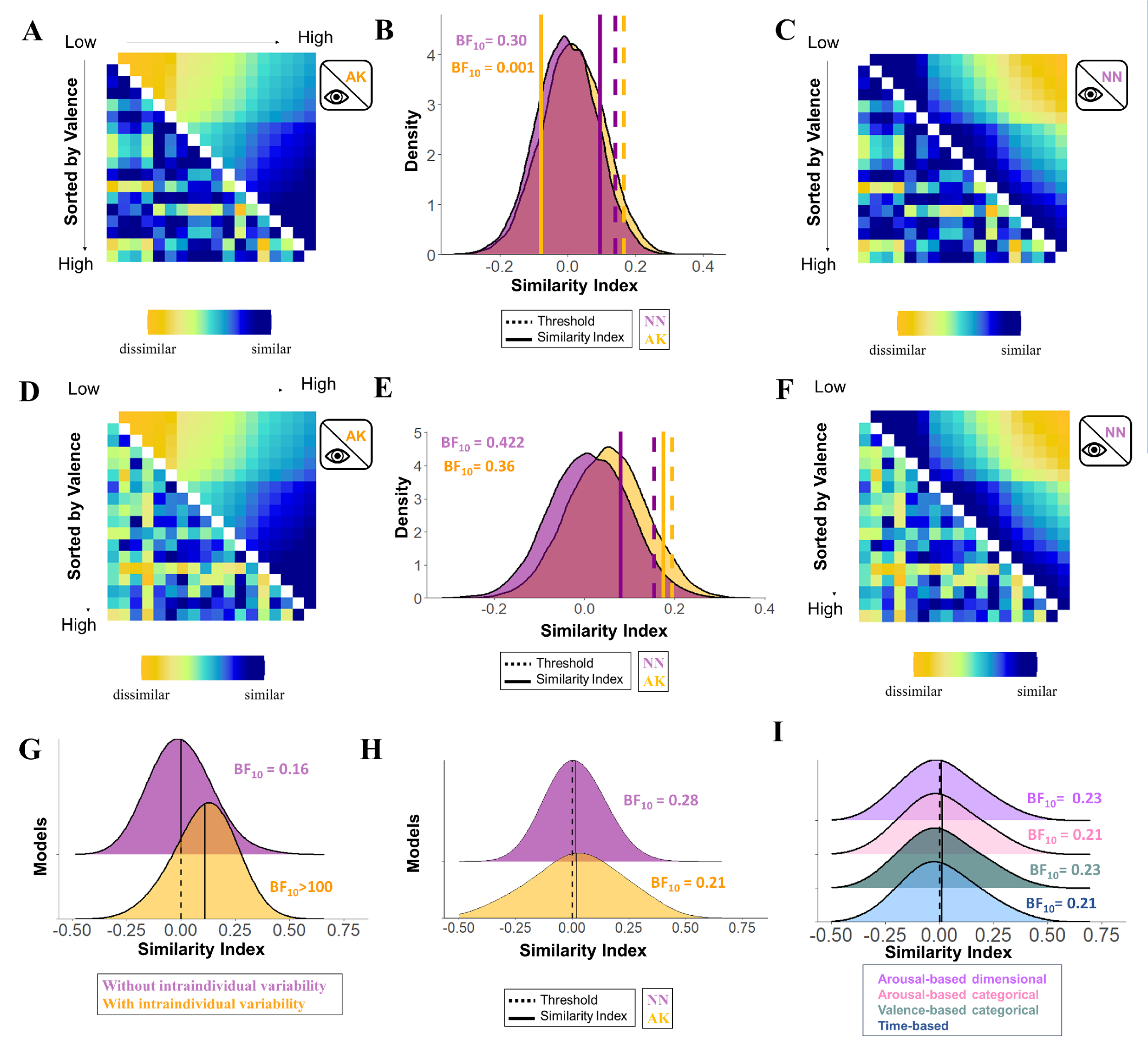


Figure S8. Association between the representational similarity matrices (RSMs) of subjective valence based on the Anna Karenina (AK) and Nearest Neighbours (NN) models (after controlling for the shared similarities), and the RSMs the startle eye blink response dismissing (A-C) and considering (D-F) intraindividual variability (replication sample, Imagery task, N= 64). A) Averaged RSM of startle dismissing intraindividual variability (lower diagonal) and averaged RSM of subjective valence based on the AK model (upper diagonal). B) Results of the permutation test. C) Averaged RSM of startle dismissing intraindividual variability (lower diagonal) and averaged RSM of subjective valence based on the NN model (upper diagonal). D) Averaged RSM of startle considering intraindividual variability (lower diagonal) and averaged RSM of subjective valence based on the AK model (upper diagonal). E) Results of the permutation test. F) Averaged RSM of startle considering intraindividual variability (lower diagonal) and averaged RSM of subjective valence based on NN model (upper diagonal). G) Distribution of the association between individual RSMs of startle with the unique contribution of the averaged RSMs of startle disregarding (purple) and considering (yellow) intraindividual variability. H) Distribution of the association between individual RSMs of startle and unique contribution of the individual RSM of valence based on the AK and NN models. I) Distribution of the association between individual RSMs of startle and individual RSMs of subjective valence based on the AK model after controlling for other models (i.e., time-based, valence-based categorical, arousal-based categorical, valence-based dimensional models).

10. Visual depiction of RSMs construction and analyses performed.


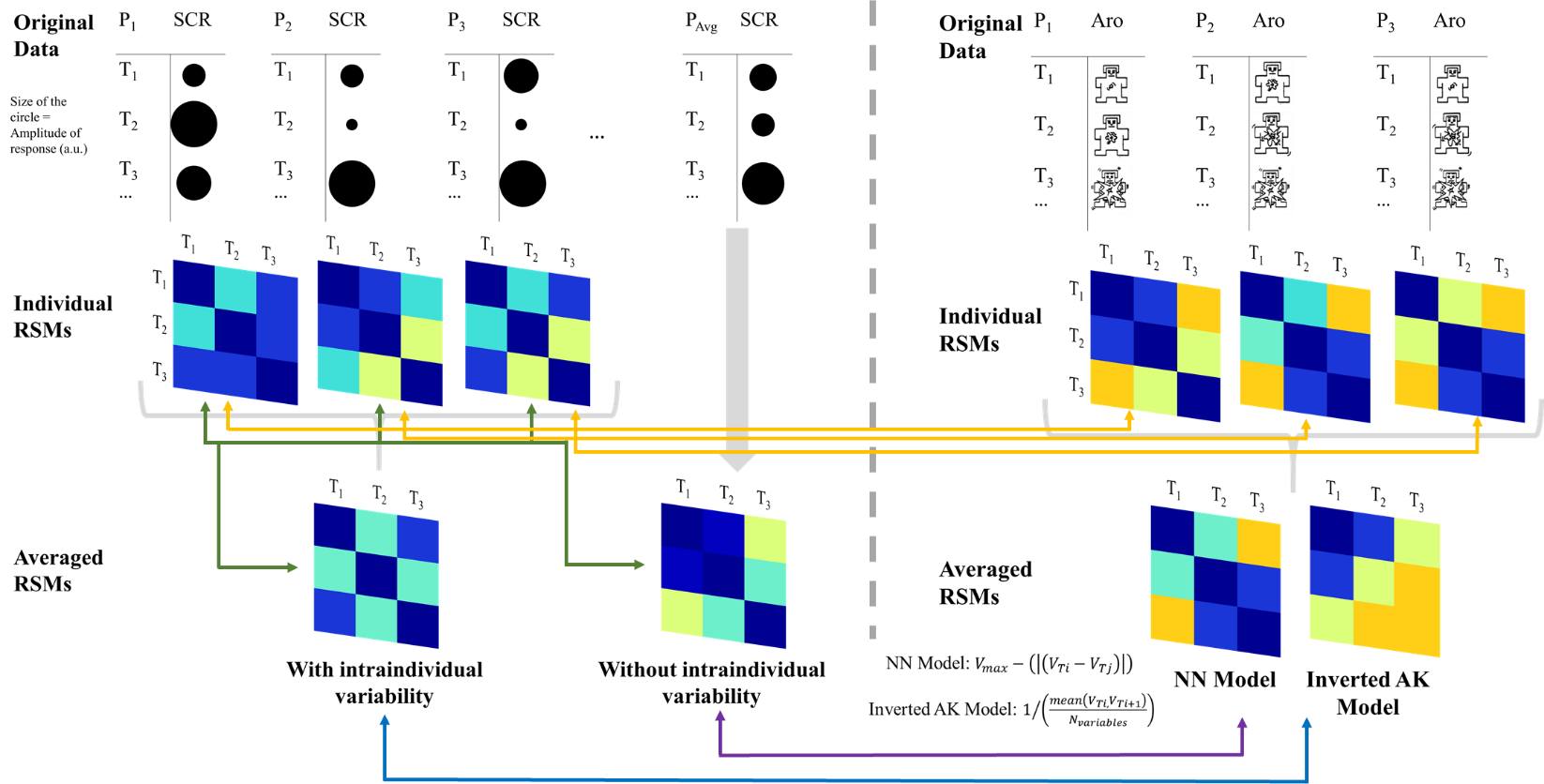


Figure S9. Visual depiction of the methodologies used and analysis performed, using the example of SCR and arousal. On the left-hand side, the step-by-step procedure to extract the individual and averaged (with and without intraindividual variability) RSMs of the physiological variable is depicted. On the right-hand side, the procedure to extract the NN and inverted AK models of affective ratings is depicted. Colored arrows indicate the analysis performed. The purple arrow depicts the association between averaged RSM disregarding intraindividual variability and the averaged RSMs of the models (e.g., NN, AK, inverted AK). The blue arrow represents the comparison between averaged RSM considering intraindividual variability and the averaged RSMs of the models. Green arrows indicate the association between individual RSMs of the physiological variable and the averaged RSMs both considering and disregarding intraindividual variability. Orange arrows depict the association between individual RSMs of physiological and subjective affective experience.

References

1. Bradley, M. M., Codispoti, M., Cuthbert, B. N. & Lang, P. J. Emotion and motivation I: Defensive and appetitive reactions in picture processing. *Emotion* **1**, 276–298 (2001).

2. Lang, P. J., Greenwald, M. K., Bradley, M. M. & Hamm, A. O. Looking at pictures: affective, facial, visceral, and behavioral reactions. *Psychophysiology* **30**, 261–273 (1993).

3. Kuhn, M. *et al.* The Neurofunctional Basis of Affective Startle Modulation in Humans: Evidence From Combined Facial Electromyography and Functional Magnetic Resonance Imaging. *Biol Psychiatry* **87**, 548–558 (2020).

4. Bates, D., Mächler, M., Bolker, B. & Walker, S. Fitting Linear Mixed-Effects Models Using lme4. *Journal of Statistical Software* **67**, 1–48 (2015).

5. Ventura-Bort, C., Wendt, J. & Weymar, M. New insights on the correspondence between subjective affective experience and physiological responses from representational similarity analysis. *Psychophysiology* **59**, e14088 (2022).
